# A depth-first search algorithm for oligonucleotide design in gene assembly

**DOI:** 10.3389/fgene.2022.1023092

**Published:** 2022-11-21

**Authors:** Hanjie Liang, Zengrui Chen, Gang Fang

**Affiliations:** Institute of Computing Science and Technology, Guangzhou University, Guangzhou, China

**Keywords:** gene assembly, depth-first search, algorithm, oligonucleotide design, melting temperature

## Abstract

When synthesizing a gene with a long DNA sequence, it is usually necessary to divide it into several fragments. Based on these fragments, a set of oligonucleotides for gene assembly is produced. Each oligonucleotide is synthesized separately by the chemical reaction, and then the obtained oligonucleotides are assembled into the full gene sequence, in a specific environment, by polymerase chain reaction (PCR) or ligase chain reaction (LCR). In this paper, an effective and efficient algorithm to divide long genes into oligonucleotide sets is presented. First, according to the length of the overlapping oligonucleotide region, the long DNA sequence to be synthesized is divided into fragments of approximately equal length. Second, the length of these fragments is iterated to dynamically optimize the length of the overlapping regions to reduce melting temperature fluctuations. Then, the improved depth-first search algorithm is used according to the design principle of pruning optimization to obtain a uniform set of oligonucleotides with very close melting temperatures. This will decrease the errors in gene assembly with PCR or LCR. Lastly, the oligonucleotides that have homologous melting temperatures needed for PCR-based synthesis and two-step assembly of the target gene are deduced and outputted.

## Introduction

Gene synthesis now mainly utilizes overlapping oligonucleotides to assemble large genes (>1000 bp) by polymerase chain reaction (PCR) or ligase chain reaction (LCR) (Stemmer et al., 1995; Au et al., 1998). To optimize the PCR or LCR process and minimize errors in assembly, gene synthesis computer programs have been developed to aid in designing the oligonucleotides. The program’s algorithm automates and streamlines the oligonucleotide design process, so that errors in assembly are minimized and large genes can be synthesized effectively. In gapless PCR assembly, some web-based applications, for example, TmPrime and DNAWorks which use an iterative algorithm, have been developed (David and DNAWorks, 2002; Marcus et al., 2009). In gapped PCR assembly, applications such as Gene2Oligo, Assembly PCR Oligo Maker, and GeneDesign that mainly rely on an iterative algorithm have been composed as well (Jean-Marie et al., 2004; Roman et al., 2005; Sarah et al., 2006). In the key step of oligonucleotide design, the input gene sequences are optimally split into oligonucleotides by the algorithm, so as to have approximately the same melting temperatures, and the overlapping regions of these oligonucleotides possess homologous melting temperatures. The best result is a deviation in melting temperature of less than 1°C in the overlapped region, which is attained with the use of a dynamic programming algorithm (Fang and Liang, 2022). However, its time complexity is too high to be used as a web-based application. Here, we present a depth-first search (DFS) algorithm with lower time complexity, for a web-based gene synthesis application, and to minimize errors in PCR assembly.

**FIGURE 1:**
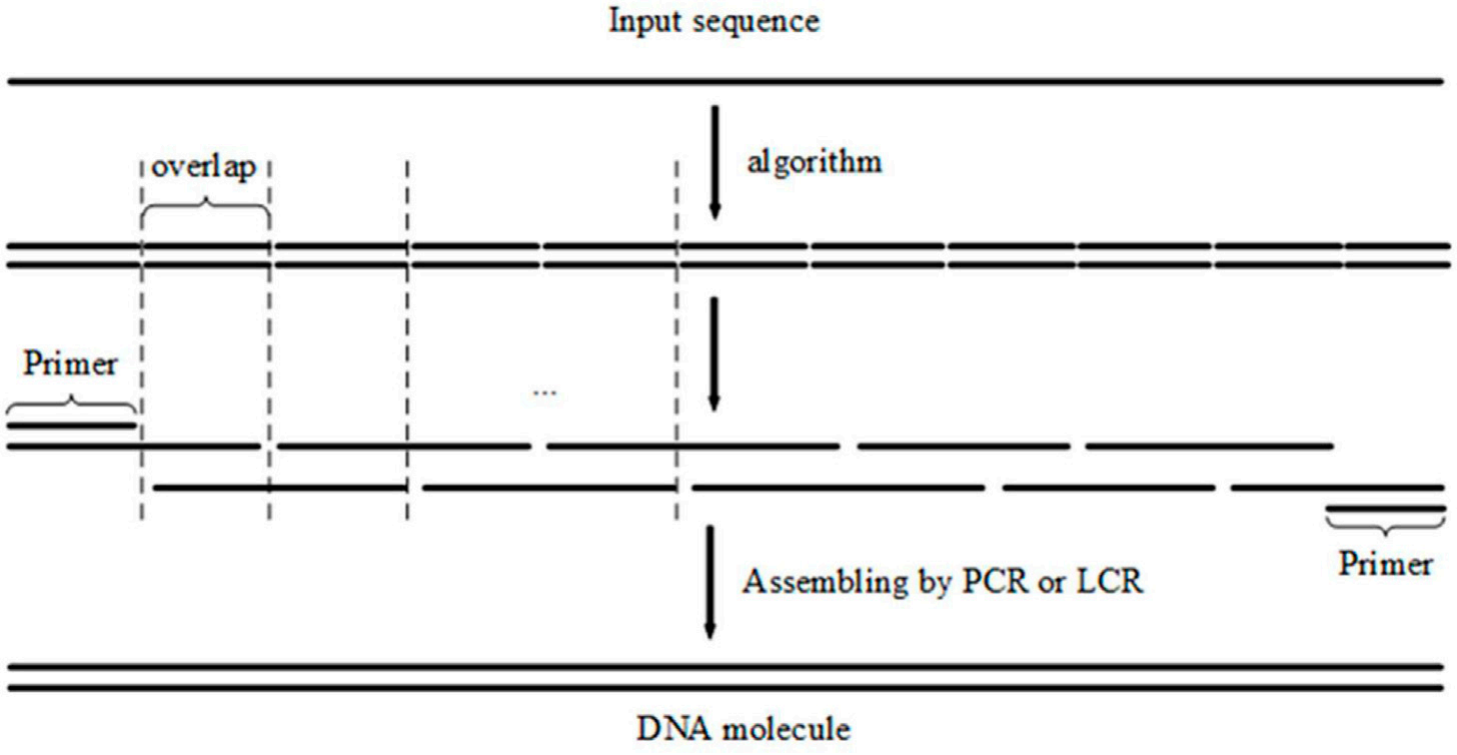
When assembling oligonucleotides with PCR or LCR, the overlapped regions of oligonucleotides are connected sequentially by the input sequence. The ‘Primer’ in the figure indicates that the primers for PCR share the same sequence with first and last overlap.

In gene synthesis, when assembling oligonucleotides with gapless PCR or LCR, all oligonucleotides are ligated tightly together with no gaps between adjacent oligonucleotides. The overlapped regions are connected sequentially according to the input sequence. Based on the aforementioned findings, the problem of splitting the input sequence into oligonucleotides with approximately the same melting temperature in overlapping regions can be treated as a problem of segmenting the input sequence into fragments with homologous melting temperatures; each fragment explicitly represents an overlapped region (Marcus et al., 2009) (Figure 1).

When assembling oligonucleotides using gapped PCR, the DNA fragments are contiguous with few base deletions. Compared with gapless PCR or LCR, gapped PCR assembly can lead to more assembly errors, but these errors are insignificant and can be ignored (Xiong et al., 2000). Gapped PCR assembly is more flexible and economical (Xiong et al., 2000). In this work, an algorithm that makes use of overlapping regions with homologous melting temperatures to output oligonucleotides for gapped PCR assembly is proposed.

## Methods

**FIGURE 2:**
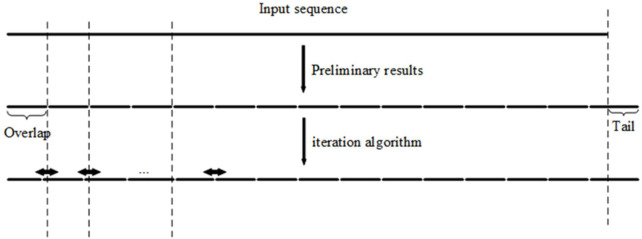
Iteration algorithm is carried out by shifting the boundary between overlaps (segments) iteratively until convergence.

According to the simple observation depicted in Figure 1, the proposed algorithm with an iteration step is introduced. First, according to the length of the overlapped regions, the long input DNA sequence to be synthesized is divided into fragments of approximately equal length. Second, we optimized the result of the initial segmentation step to reduce fluctuations in the melting temperatures of the overlapped regions (Figure 2). The input DNA sequence is processed by the iteration algorithm to split it into segments with similar melting temperatures. By iteratively adjusting the boundary between segments obtained from the initial segmented result, the melting temperature of the segments is first calculated by the algorithm and then the segments with the closest melting temperatures are selected for combination. In Table 1, the iteration algorithm is given by detailed pseudocode.

**TABLE 1:** Details of the iteration algorithm. The iteration algorithm is an initial algorithm that roughly segments the input sequence.

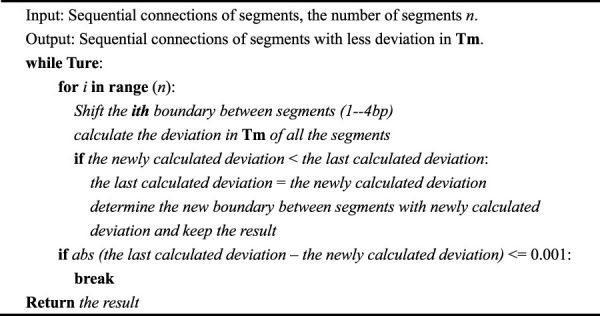

**FIGURE 3:**
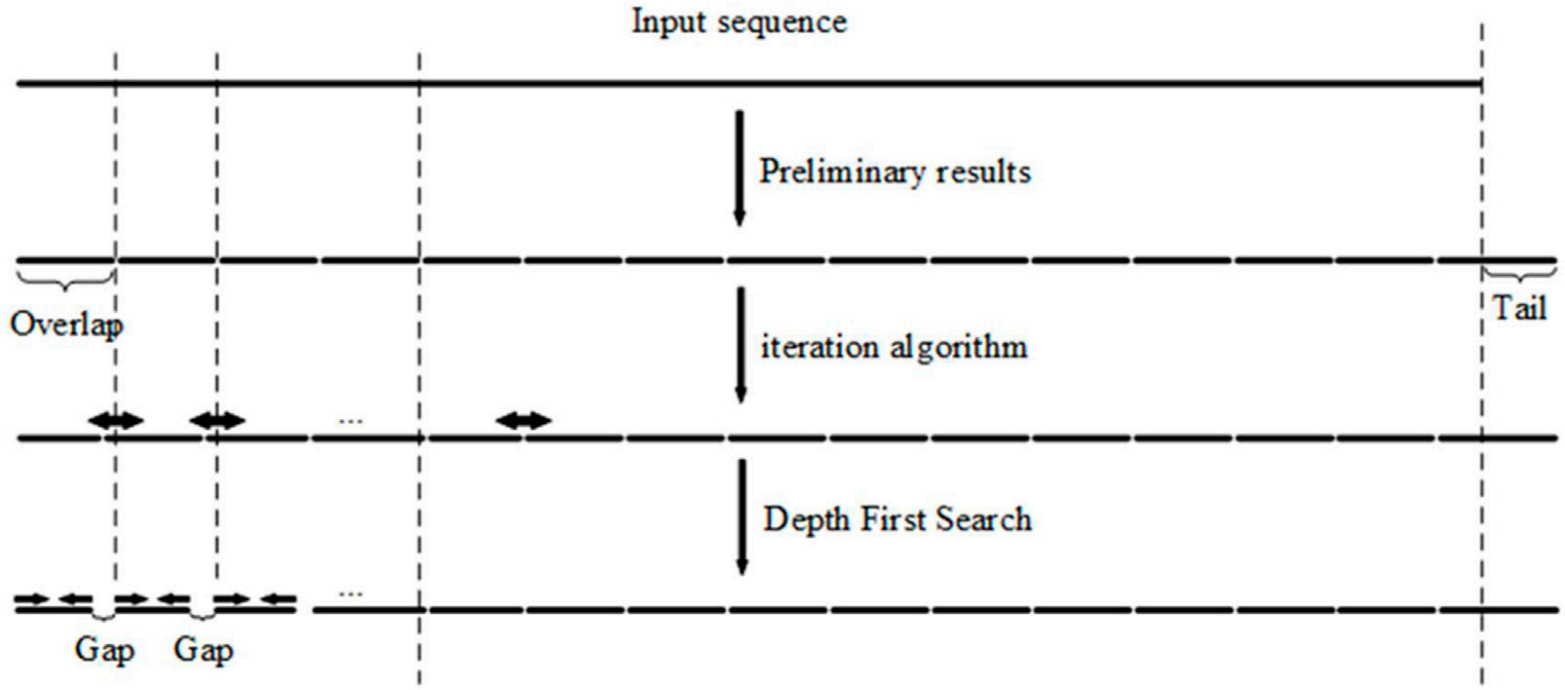
Depth-first search algorithm takes the results from the iteration algorithm, shrinks each end of an overlap, and produces a gap between segments. This reduces the standard deviation of the overlap melting temperatures.

A nearest neighbor model is used to calculate melting temperatures along with Santa Lucia’s thermodynamic parameters (Santa Lucia and Hicks, 2004), the salt and oligonucleotide concentrations, and the totality of phosphates in the duplex (Owczarzy et al., 2008). The equations and procedures used to calculate DNA melting temperature are described in detail in the Supplementary Material. After processing with the iteration algorithm, the oligonucleotide set can be deduced from the segmentation results. These oligonucleotides can be used for LCR or gapless PCR assembly; however, this step may not provide the best solution to the problem. In an attempt to further reduce the melting temperature fluctuation of overlapped regions, we present a DFS algorithm, which serves as the foundation for a large number of graphic applications that utilize tree-based search algorithms. Given a vertex, the DFS algorithm can find all reachable vertices as well as directed and undirected graphs containing the shortest paths from one vertex to others (Cong, 2010). As an important graph-search algorithm, the DFS algorithm plays an important role in solving issues in computer science like planarity testing, scheduling problems, inspecting network structuring, and topological sorting (Palanisamy and Vijayanathan, 2020). In our work, the DFS algorithm was adapted to solve the oligonucleotides design problem in gene assembly (Figure 3). In Table 2, the DFS algorithm adapted to minimize the fluctuation of melting temperatures of oligonucleotide overlapped region is shown in detailed pseudocode.

**TABLE 2:** Depth-first search algorithm (DFS).

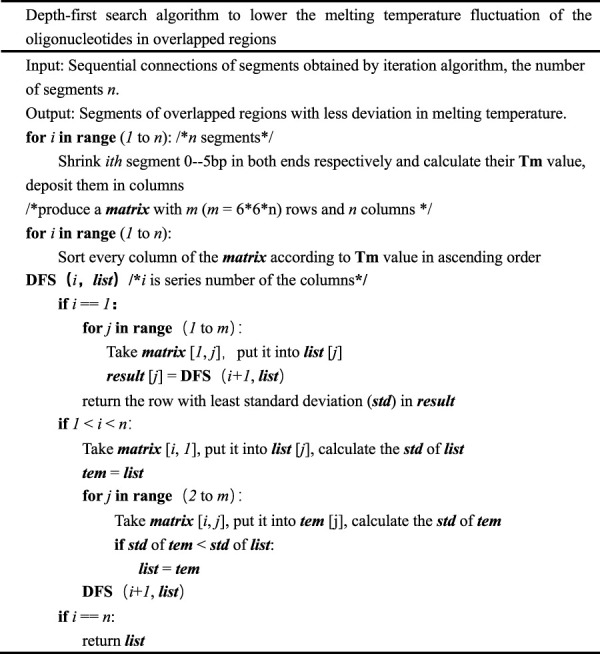

Oligonucleotides with close, uniform melting temperatures can be generated using results computed by the DFS algorithm for gapped PCR assembly. A short tail may be appended to the 3’ end of an input sequence (Figures 2, 3), which will guarantee the imbricated structure of the oligonucleotides output in the PCR reaction diagrammed in Figure 1. The added tail can be removed by PCR using specific primers.

**TABLE 3:** Oligonucleotide set designed by three algorithms. Compared to other algorithms, the depth-first search algorithm does not surpass the integrated algorithm, but it takes less time to process. For a fair comparison, the algorithms were tested under the same conditions.

Algorithm	Gene		
	S100A4 (752 bp) overlap Tm std	PKB2 (1446 bp) overlap Tm std	GFPuv (760 bp) overlap Tm std
TmPrime	1.14	1.23	0.93
Depth-first search	0.84	0.52	0.40
Integrated algorithm (gapped)	0.27	0.49	0.17

## Results

It is crucial to ensure uniformity of melting temperature, especially in the overlapped oligo regions, when designing oligonucleotides for gene synthesis. It will reduce mis-hybridization between oligonucleotides and decrease errors in assembly. The oligonucleotide sets produced by the presented algorithm cannot achieve a smaller standard deviation in overlap melting temperatures than the result produced by the integrated algorithm, but the result is acceptable for gapped PCR assembly and results in a lower SD of Tm in the overlapped regions than TmPrime (Table 3). In fact, there are few base deletions (gaps) and frequently no gaps between adjacent oligonucleotides such that the final oligonucleotides are contiguous in gapped assembly. This phenomenon is caused by the algorithm’s intrinsic nature. The number of bases to be shrunk in the first *for* loop in the DFS algorithm (Table 2) can be changed to modify the number of gaps. Readers are referred to Supplementary Material or https://github.com/Jacka03/oligoOptimizer for more information. *Python* 3.7 was adopted for the algorithm implementation. On a desktop computer with dual 3.3-GHz Intel Xeons and 4 GB RAM, it takes less than 2 s to design a set of oligonucleotides for a multi-kilobase (<3 kb) gene. Under the same conditions, the integrated algorithm takes 10s (Fang and Liang, 2022). The depth-first search algorithm was developed for web-based application, which needs to return a result with no unacceptable delay.

## Discussion

In this paper, we presented an effective and efficient depth-first search algorithm for oligonucleotide design in gene synthesis based on its intrinsic nature. The average melting temperature of the final oligonucleotide set can be used to set the annealing temperature of assembly. It is difficult to calculate the time complexity of an iteration algorithm, but the variance threshold between the last calculated deviation and the newly calculated deviation can be adjusted to achieve rapid convergence. Empirically, a variance threshold of 0.001 often yields a rapid convergence. Given the number of sequential connections of segments, *L*, along with the number of possible column segments, *N*, the time complexity is *O*(*LN* ^*3*^) for the dynamic programming algorithm and *O*(*N* ^*L*^) for the exhaustive algorithm (Fang and Liang, 2022). In contrast, the time complexity is *O*(*N* ^*2*^) for the DFS algorithm, which will guarantee the technical feasibility of this algorithm. Furthermore, it only requires a computer of average speed and RAM for running, which is more suitable for a web-based application needed to return a result with little delay. Based on the simple observation, gapless assembly can use an iteration algorithm to generate the oligonucleotides, regardless of its elementary results. Theoretically, this kind of optimization problem is not guaranteed to have the best solution; and even if there is one, it is difficult to acquire (Pevzner and Waterman, 1995; Berman et al., 2002). With regard to this problem, the presented depth-first algorithm is a type of approximation algorithm, which means that it runs faster than an integrative algorithm. When it is carried out after an iteration algorithm, an acceptable result is acquired. The oligonucleotides produced have a greater uniformity of melting temperature than TmPrime, which reduces assembly error; but these oligonucleotides can only be used for gapped PCR assembly, which can result in a higher assembly error rate than gapless assembly. The oligonucleotides produced by the DFS algorithm are very close in Tm, which can diminish the effect caused by gaps between adjacent oligonucleotides. Synchronization of the melting temperatures of the overlapped region is the vital factor in gene assembly, and it is important to take into account factors like the appearance of repeated regions, high CG content regions, etc. These factors can cause mis-hybridization between oligonucleotides with the formation of unwanted secondary structures that can increase errors in the PCR reaction. In this paper, we stress the importance of Tm uniformity of overlapped regions as a way to compensate for potential mismatch problems and facilitate hybridization between overlapping oligonucleotides. The reason for this is that oligonucleotides with uniform Tm are more likely to simultaneously overlap and hybridize correctly under the same temperature.

The position and number of bases to be shrunk in the first *for* loop in the DFS algorithm (Table 2) can be changed to modify the position and number of gaps in the algorithm. The size of segments to be split before using the iteration algorithm can also be changed to modify the length of oligonucleotides (in general, a base number of 20–30 bp to be split will generate an overlap of 20–30 bp and oligonucleotides of 40–60 bp in length). The DFS algorithm is the core of this project. While the input sequence is only approximately segmented by iteration algorithms, the uniformity of melting temperature of oligonucleotides for gene synthesis is further achieved by the DFS algorithm, a strategy that is widely adopted in the optimization theory (Hidayatullah et al., 2017; Zheng et al., 2021). It is expected to experience an explosive increase in application due to its practicability and universality in biology, especially in synthetic biology. The integrated algorithm in our previous study was complex, consisting of three main sub-algorithms (the greedy algorithm, iteration algorithm, and dynamic programming algorithm) (Fang and Liang, 2022). In theory, its time complexity is *O*(*LN* ^*3*^), but in practice it is variable. When processing longer sequences and for more accurate results, the time complexity always exceeded *O*(*LN* ^*3*^) and required a more powerful computer to run; furthermore, it is not open-source. Although DFS for this application is an approximation algorithm, it is open-source. It was not only developed for web-based application, but also for people in related fields to modify and develop related algorithms, and it is easy to carry out on an ordinary desktop computer. Oligo design is very important in gene synthesis. The web-based applications, TmPrime, DNAWorks, Gene2Oligo, Assembly PCR Oligo Maker etc., are no longer available and their source code is not always open-source. The DFS algorithm has been developed for oligo design and is open-source for all users. This project has been written into the computer program to facilitate gene synthesis.

## Data Availability

The original contributions presented in the study are included in the article/Supplementary Material; further inquiries can be directed to the corresponding author.
