# Supplementary material for: A depth-first search algorithm for oligonucleotide design in gene assembly
Source: Front Genet. 2022 Nov 21;13:1023092. doi: 10.3389/fgene.2022.1023092 (PMC9720172; doi:10.3389/fgene.2022.1023092)
Supplement: Supplementary file 1 [file Table1.DOCX]

**Supplementary Materials**

E. coli codon-optimized GFPuv 760bp

AGAGGATCCCCGGGTACCGGTAGAAAAAATGAGTAAAGGAGAAGAACTTTTCACTGGAGTTGTCCCAATTCTTGTTGAATTAGATGGTGATGTTAACGGGCACAAATTTTCTGTCAGTGGAGAGGGTGAAGGTGATGCAACATACGGAAAACTTACCCTTAAATTTATTTGCACTACTGGAAAACTACCTGTTCCATGGCCAACACTTGTCACTACTTTCTCTTATGGTGTTCAATGCTTTTCCCGTTATCCGGATCATATGAAACGGCATGACTTTTTCAAGAGTGCCATGCCCGAAGGTTATGTACAGGAACGCACTATATCTTTCAAAGATGACGGGAACTACAAGACGCGTGCTGAAGTCAAGTTTGAAGGTGATACCCTTGTTAATCGTATCGAGTTAAAAGGTATTGATTTTAAAGAAGATGGAAACATTCTCGGACACAAACTCGAGTACAACTATAACTCACACAATGTATACATCACGGCAGACAAACAAAAGAATGGAATCAAAGCTAACTTCAAAATTCGCCACAACATTGAAGATGGAAGCGTTCAACTAGCAGACCATTATCAACAAAATACTCCAATTGGCGATGGCCCTGTCCTTTTACCAGACAACCATTACCTGTCGACACAATCTGCCCTTTCGAAAGATCCCAACGAAAAGCGTGACCACATGGTCCTTCTTGAGTTTGTAACTGCTGCTGGGATTACACATGGCATGGATGAGCTCTACAAATAATGAATTCCAACTGAG

E. coli codon-optimized PKB2 1446bp

ATGAATGAGGTGTCTGTCATCAAAGAAGGCTGGCTCCACAAGCGTGGTGAATACATCAAGACCTGGAGGCCACGGTACTTCCTGCTGAAGAGCGACGGCTCCTTCATTGGGTACAAGGAGAGGCCCGAGGCCCCTGATCAGACTCTACCCCCCTTAAACAACTTCTCCGTAGCAGAATGCCAGCTGATGAAGACCGAGAGGCCGCGACCCAACACCTTTGTCATACGCTGCCTGCAGTGGACCACAGTCATCGAGAGGACCTTCCACGTGGATTCTCCAGACGAGAGGGAGGAGTGGATGCGGGCCATCCAGATGGTCGCCAACAGCCTCAAGCAGCGGGCCCCAGGCGAGGACCCCATGGACTACAAGTGTGGCTCCCCCAGTGACTCCTCCACGACTGAGGAGATGGAAGTGGCGGTCAGCAAGGCACGGGCTAAAGTGACCATGAATGACTTCGACTATCTCAAACTCCTTGGCAAGGGAACCTTTGGCAAAGTCATCCTGGTGCGGGAGAAGGCCACTGGCCGCTACTACGCCATGAAGATCCTGCGAAAGGAAGTCATCATTGCCAAGGATGAAGTCGCTCACACAGTCACCGAGAGCCGGGTCCTCCAGAACACCAGGCACCCGTTCCTCACTGCGCTGAAGTATGCCTTCCAGACCCACGACCGCCTGTGCTTTGTGATGGAGTATGCCAACGGGGGTGAGCTGTTCTTCCACCTGTCCCGGGAGCGTGTCTTCACAGAGGAGCGGGCCCGGTTTTATGGTGCAGAGATTGTCTCGGCTCTTGAGTACTTGCACTCGCGGGACGTGGTATACCGCGACATCAAGCTGGAAAACCTCATGCTGGACAAAGATGGCCACATCAAGATCACTGACTTTGGCCTCTGCAAAGAGGGCATCAGTGACGGGGCCACCATGAAAACCTTCTGTGGGACCCCGGAGTACCTGGCGCCTGAGGTGCTGGAGGACAATGACTATGGCCGGGCCGTGGACTGGTGGGGGCTGGGTGTGGTCATGTACGAGATGATGTGCGGCCGCCTGCCCTTCTACAACCAGGACCACGAGCGCCTCTTCGAGCTCATCCTCATGGAAGAGATCCGCTTCCCGCGCACGCTCAGCCCCGAGGCCAAGTCCCTGCTTGCTGGGCTGCTTAAGAAGGACCCCAAGCAGAGGCTTGGTGGGGGGCCCAGCGATGCCAAGGAGGTCATGGAGCACAGGTTCTTCCTCAGCATCAACTGGCAGGACGTGGTCCAGAAGAAGCTCCTGCCACCCTTCAAACCTCAGGTCACGTCCGAGGTCGACACAAGGTACTTCGATGATGAATTTACCGCCCAGTCCATCACAATCACACCCCCTGACCGCTATGACAGCCTGGGCTTACTGGAGCTGGACCAGCGGACCCACTTCCCCCAGTTCTCCTACTCGGCCAGCATCCGCGAGTGA

S100A4 gene 752bp

GTTTTTGTTTCTGAATCTTTATTTTTTTAAGAGACAAGGTCCTCTGTGTTGCTCAGGCTGGAGAGCAGTGGCTTGAGCATAGCCAACTGCAGTCTCGAACTCCTGGGCTCAAATGATCCTCCTGTCTCAGCTTCCTGACTAGCTGGGACTACAGGCTACAGCCATGCTGCCCAGCTAATTAAAAAAAAAAATTGTTTTTCCTTTTTATAGAGACAGAAGTCTCTCTATGTTGCCTAGGCTGGTCTTGAACTCCTGGCCTCAGGCGATCCTCCCATCTCCCCCCTAGCTTTTGTGTCACCACATTTCCAGGGCAATCTCCCACCTGTCACCCACCACCCCCTGCATCTCCTTTCCTAGGTCCCCATGGGACTACTCCCTGTCCCCCATGCTCCAGGCACAGGCTGCCCCTTCCTCCACCTCTCTAAAACTCAGGCTGAGCTATGTACACTGGGTGGTGCCCATCTCATCCAGTCCCCTGCTAGTAACCGCTAGGGCTTACCCGTTACCCACGGGTGCCCACCTGGGAACAGGAGGCTTGGTTCCACGGCTGGGCTGGTGGAGGGTGCTGTGGCACTTACCGCATCAGCCCACAGCAGGAAGGCAGTATCCGCTCTCCCCTGTCCCCTGCTATGGGCAGGGCCTGGCTGGGGTATAAATAGGTCAGACCTCTGGGCCGTCCCCATTCTTCCCCTCTCTACAACCCTCTCTCCTCAGCGCTTCTTCATCAAGATCTGGCCTCGGCGGCCAAGCTT

**Table S1.** The parameters for designing oligonucleotides for the three genes assembly

| The parameters include all the factors that a real-time PCR need |
| --- |
| Na^+^/K^+^ ion concentration: 50mM;  Mg^2+^ ion concentration: 8mM;  dNTP concentration: 4mM;  Tris^2+^ concentration: 10mM;  oligonucleotide concentration: 10nM;  primer concentration: 400nM; |

**Table S2.** Oligonucleotides set designed by integrated algorithm for *E. coli* codon-optimized PKB2 (for gapped assembly).

| label | Oligonucleotide sequence (5’ to 3’) | len | Tm | overlap |
| --- | --- | --- | --- | --- |
| F0 | GAATGAGGTGTCTGTCATCAAAGAAGGCTGGCTCCACAAGCGTGGTGAAT | 50 | 64.04 | 22 |
| R0 | CGTGGCCTCCAGGTCTTGATGTATTCACCACGCTTGTGGAGCCA | 44 | 64.03 | 22 |
| F1 | ACATCAAGACCTGGAGGCCACGGTACTTCCTGCTGAAGAGCGACG | 45 | 63.19 | 23 |
| R1 | CCTCTCCTTGTACCCAATGAAGGAGCCGTCGCTCTTCAGCAGGAAGTAC | 49 | 65.2 | 26 |
| F2 | GCTCCTTCATTGGGTACAAGGAGAGGCCCGAGGCCCCTGATCAGACT | 47 | 64.96 | 21 |
| R2 | ACGGAGAAGTTGTTTAAGGGGGGTAGAGTCTGATCAGGGGCCTCGGG | 47 | 64.28 | 26 |
| F3 | CTACCCCCCTTAAACAACTTCTCCGTAGCAGAATGCCAGCTGATGAAGACC | 51 | 65.27 | 25 |
| R3 | GTGTTGGGTCGCGGCCTCTCGGTCTTCATCAGCTGGCATTCTGCT | 45 | 65.35 | 20 |
| F4 | GAGAGGCCGCGACCCAACACCTTTGTCATACGCTGCCTGCAGTG | 44 | 64.66 | 24 |
| R4 | GAAGGTCCTCTCGATGACTGTGGTCCACTGCAGGCAGCGTATGACAAAG | 49 | 64.64 | 25 |
| F5 | GACCACAGTCATCGAGAGGACCTTCCACGTGGATTCTCCAGACGAGAGG | 49 | 64.67 | 24 |
| R5 | GATGGCCCGCATCCACTCCTCCCTCTCGTCTGGAGAATCCACGTG | 45 | 64.95 | 21 |
| F6 | GAGGAGTGGATGCGGGCCATCCAGATGGTCGCCAACAGCCTCAA | 44 | 65.0 | 23 |
| R6 | GCCTGGGGCCCGCTGCTTGAGGCTGTTGGCGACCATCTG | 39 | 65.21 | 16 |
| F7 | GCAGCGGGCCCCAGGCGAGGACCCCATGGACTACAAGTGTG | 41 | 64.91 | 25 |
| R7 | GGAGGAGTCACTGGGGGAGCCACACTTGTAGTCCATGGGGTCCTC | 45 | 63.42 | 20 |
| F8 | GCTCCCCCAGTGACTCCTCCACGACTGAGGAGATGGAAGTGGC | 43 | 63.78 | 23 |
| R8 | AGCCCGTGCCTTGCTGACCGCCACTTCCATCTCCTCAGTCGT | 42 | 64.34 | 19 |
| F9 | GGTCAGCAAGGCACGGGCTAAAGTGACCATGAATGACTTCGACTATCTCAA | 51 | 64.83 | 32 |
| R9 | CCAAAGGTTCCCTTGCCAAGGAGTTTGAGATAGTCGAAGTCATTCATGGTCACTTT | 56 | 65.02 | 24 |
| F10 | ACTCCTTGGCAAGGGAACCTTTGGCAAAGTCATCCTGGTGCGGGAGAA | 48 | 64.75 | 24 |
| R10 | CGTAGTAGCGGCCAGTGGCCTTCTCCCGCACCAGGATGACTTTG | 44 | 64.87 | 20 |
| F11 | GGCCACTGGCCGCTACTACGCCATGAAGATCCTGCGAAAGGAAGTCA | 47 | 65.16 | 27 |
| R11 | GTGAGCGACTTCATCCTTGGCAATGATGACTTCCTTTCGCAGGATCTTCATGG | 53 | 64.71 | 25 |
| F12 | CATTGCCAAGGATGAAGTCGCTCACACAGTCACCGAGAGCCGGGTC | 46 | 65.57 | 21 |
| R12 | CGGGTGCCTGGTGTTCTGGAGGACCCGGCTCTCGGTGACTGT | 42 | 65.02 | 21 |
| F13 | CTCCAGAACACCAGGCACCCGTTCCTCACTGCGCTGAAGTATGCC | 45 | 64.99 | 24 |
| R13 | AGGCGGTCGTGGGTCTGGAAGGCATACTTCAGCGCAGTGAGGAA | 44 | 64.6 | 20 |
| F14 | TTCCAGACCCACGACCGCCTGTGCTTTGTGATGGAGTATGCCAACG | 46 | 65.09 | 26 |
| R14 | CAGGTGGAAGAACAGCTCACCCCCGTTGGCATACTCCATCACAAAGCAC | 49 | 65.21 | 23 |
| F15 | GGGGTGAGCTGTTCTTCCACCTGTCCCGGGAGCGTGTCTTCACA | 44 | 64.79 | 21 |
| R15 | ATAAAACCGGGCCCGCTCCTCTGTGAAGACACGCTCCCGGGA | 42 | 64.18 | 21 |
| F16 | GAGGAGCGGGCCCGGTTTTATGGTGCAGAGATTGTCTCGGCTCTT | 45 | 64.51 | 24 |
| R16 | CGTCCCGCGAGTGCAAGTACTCAAGAGCCGAGACAATCTCTGCACC | 46 | 65.42 | 22 |
| F17 | GAGTACTTGCACTCGCGGGACGTGGTATACCGCGACATCAAGCTGG | 46 | 64.58 | 24 |
| R17 | GCCATCTTTGTCCAGCATGAGGTTTTCCAGCTTGATGTCGCGGTATACCA | 50 | 64.32 | 26 |
| F18 | AAAACCTCATGCTGGACAAAGATGGCCACATCAAGATCACTGACTTTGGCCTCT | 54 | 65.29 | 28 |
| R18 | CCCGTCACTGATGCCCTCTTTGCAGAGGCCAAAGTCAGTGATCTTGATGTG | 51 | 65.99 | 23 |
| F19 | GCAAAGAGGGCATCAGTGACGGGGCCACCATGAAAACCTTCTGTGGG | 47 | 64.48 | 24 |
| R19 | GCGCCAGGTACTCCGGGGTCCCACAGAAGGTTTTCATGGTGGC | 43 | 65.57 | 19 |
| F20 | ACCCCGGAGTACCTGGCGCCTGAGGTGCTGGAGGACAATGACTATG | 46 | 65.34 | 27 |
| R20 | CCAGTCCACGGCCCGGCCATAGTCATTGTCCTCCAGCACCTCAG | 44 | 64.69 | 17 |
| F21 | GCCGGGCCGTGGACTGGTGGGGGCTGGGTGTGGTCAT | 37 | 64.71 | 20 |
| R21 | GCGGCCGCACATCATCTCGTACATGACCACACCCAGCCCCCA | 42 | 64.94 | 20 |
| F22 | ACGAGATGATGTGCGGCCGCCTGCCCTTCTACAACCAGGACCAC | 44 | 65.17 | 24 |
| R22 | AGGATGAGCTCGAAGAGGCGCTCGTGGTCCTGGTTGTAGAAGGGCAG | 47 | 64.93 | 22 |
| F23 | AGCGCCTCTTCGAGCTCATCCTCATGGAAGAGATCCGCTTCCCGCG | 46 | 65.15 | 21 |
| R23 | GCCTCGGGGCTGAGCGTGCGCGGGAAGCGGATCTCTTCC | 39 | 64.96 | 18 |
| F24 | CACGCTCAGCCCCGAGGCCAAGTCCCTGCTTGCTGGGCT | 39 | 64.6 | 21 |
| R24 | GCCTCTGCTTGGGGTCCTTCTTAAGCAGCCCAGCAAGCAGGGACTTG | 47 | 64.33 | 24 |
| F25 | TTAAGAAGGACCCCAAGCAGAGGCTTGGTGGGGGGCCCAGCG | 42 | 64.1 | 16 |
| R25 | GTGCTCCATGACCTCCTTGGCATCGCTGGGCCCCCCACC | 39 | 64.63 | 23 |
| F26 | ATGCCAAGGAGGTCATGGAGCACAGGTTCTTCCTCAGCATCAACTGGCA | 49 | 65.18 | 25 |
| R26 | GGAGCTTCTTCTGGACCACGTCCTGCCAGTTGATGCTGAGGAAGAACC | 48 | 64.94 | 23 |
| F27 | GGACGTGGTCCAGAAGAAGCTCCTGCCACCCTTCAAACCTCAGGTC | 46 | 64.48 | 23 |
| R27 | GTACCTTGTGTCGACCTCGGACGTGACCTGAGGTTTGAAGGGTGGCA | 47 | 64.83 | 23 |
| F28 | CGTCCGAGGTCGACACAAGGTACTTCGATGATGAATTTACCGCCCAGTCC | 50 | 65.04 | 26 |
| R28 | CGGTCAGGGGGTGTGATTGTGATGGACTGGGCGGTAAATTCATCATCGA | 49 | 64.31 | 23 |
| F29 | ATCACAATCACACCCCCTGACCGCTATGACAGCCTGGGCTTACTGGAG | 48 | 64.78 | 25 |
| R29 | GAAGTGGGTCCGCTGGTCCAGCTCCAGTAAGCCCAGGCTGTCATAG | 46 | 64.76 | 21 |
| F30 | CTGGACCAGCGGACCCACTTCCCCCAGTTCTCCTACTCGGCCA | 43 | 65.11 | 22 |
| R30 | TGGCCGAGTAGGAGAACTGGGG | 22 | 67.8 |  |
| F_Primer | GAATGAGGTGTCTGTCATCAAAGAAGGC | 28 | 63.6 |  |
| R_Primer | TGGCCGAGTAGGAGAACTGGGG | 22 | 67.8 |  |
|  |  |  |  |  |

**Table S3.** Oligonucleotides set designed by depth-first search algorithm for *E. coli* codon-optimized PKB2 (red letters are added tail).

| **index** | **oligos** | **tm** | **overlap** | **length** |
| --- | --- | --- | --- | --- |
| F0 | ATGAATGAGGTGTCTGTCATCAAAGAAGGCTGGCTCCACAAGCGTGGTGAATACA | 65.62 | 24 | 55 |
| R0 | GTACCGTGGCCTCCAGGTCTTGATGTATTCACCACGCTTGTGGAGCC | 66.62 | 23 | 47 |
| F1 | TCAAGACCTGGAGGCCACGGTACTTCCTGCTGAAGAGCGACGGCT | 66.41 | 22 | 45 |
| R1 | GGCCTCTCCTTGTACCCAATGAAGGAGCCGTCGCTCTTCAGCAGGAA | 65.81 | 25 | 47 |
| F2 | CCTTCATTGGGTACAAGGAGAGGCCCGAGGCCCCTGATCAGACTCTACC | 66.79 | 24 | 49 |
| R2 | CATTCTGCTACGGAGAAGTTGTTTAAGGGGGGTAGAGTCTGATCAGGGGCCTCG | 65.9 | 30 | 54 |
| F3 | CCCCTTAAACAACTTCTCCGTAGCAGAATGCCAGCTGATGAAGACCGAGAGGC | 65.86 | 23 | 53 |
| R3 | CGTATGACAAAGGTGTTGGGTCGCGGCCTCTCGGTCTTCATCAGCTGG | 66.83 | 25 | 48 |
| F4 | CGCGACCCAACACCTTTGTCATACGCTGCCTGCAGTGGACCACAGTC | 66.53 | 22 | 47 |
| R4 | GAATCCACGTGGAAGGTCCTCTCGATGACTGTGGTCCACTGCAGGCAG | 66.23 | 26 | 48 |
| F5 | ATCGAGAGGACCTTCCACGTGGATTCTCCAGACGAGAGGGAGGAGTGGAT | 66.27 | 24 | 50 |
| R5 | CGACCATCTGGATGGCCCGCATCCACTCCTCCCTCTCGTCTGGA | 66.91 | 20 | 44 |
| F6 | GCGGGCCATCCAGATGGTCGCCAACAGCCTCAAGCAGCGGG | 67.51 | 21 | 41 |
| R6 | GGGGTCCTCGCCTGGGGCCCGCTGCTTGAGGCTGTTGG | 66.18 | 17 | 38 |
| F7 | CCCCAGGCGAGGACCCCATGGACTACAAGTGTGGCTCCCCCA | 66.76 | 23 | 42 |
| R7 | CATCTCCTCAGTCGTGGAGGAGTCACTGGGGGAGCCACACTTGTAGTCC | 66.65 | 26 | 49 |
| F8 | GTGACTCCTCCACGACTGAGGAGATGGAAGTGGCGGTCAGCAAGGCA | 66.23 | 21 | 47 |
| R8 | TCGAAGTCATTCATGGTCACTTTAGCCCGTGCCTTGCTGACCGCCACTTC | 66.8 | 29 | 50 |
| F9 | CGGGCTAAAGTGACCATGAATGACTTCGACTATCTCAAACTCCTTGGCAAGGGAACCT | 66.51 | 29 | 58 |
| R9 | CCGCACCAGGATGACTTTGCCAAAGGTTCCCTTGCCAAGGAGTTTGAGATAG | 66.24 | 23 | 52 |
| F10 | TTGGCAAAGTCATCCTGGTGCGGGAGAAGGCCACTGGCCGCTAC | 66.48 | 21 | 44 |
| R10 | TCCTTTCGCAGGATCTTCATGGCGTAGTAGCGGCCAGTGGCCTTCTC | 66.4 | 25 | 47 |
| F11 | ACGCCATGAAGATCCTGCGAAAGGAAGTCATCATTGCCAAGGATGAAGTCGCT | 66.29 | 28 | 53 |
| R11 | CCGGCTCTCGGTGACTGTGTGAGCGACTTCATCCTTGGCAATGATGACT | 66.18 | 21 | 49 |
| F12 | CACACAGTCACCGAGAGCCGGGTCCTCCAGAACACCAGGCACC | 66.18 | 22 | 43 |
| R12 | GCATACTTCAGCGCAGTGAGGAACGGGTGCCTGGTGTTCTGGAGGAC | 66.64 | 25 | 47 |
| F13 | CGTTCCTCACTGCGCTGAAGTATGCCTTCCAGACCCACGACCGCC | 66.45 | 20 | 45 |
| R13 | CGTTGGCATACTCCATCACAAAGCACAGGCGGTCGTGGGTCTGGAAG | 65.94 | 27 | 47 |
| F14 | TGTGCTTTGTGATGGAGTATGCCAACGGGGGTGAGCTGTTCTTCCACCTG | 66.18 | 23 | 50 |
| R14 | TGTGAAGACACGCTCCCGGGACAGGTGGAAGAACAGCTCACCCC | 65.97 | 21 | 44 |
| F15 | TCCCGGGAGCGTGTCTTCACAGAGGAGCGGGCCCGGTTTTATG | 66.82 | 22 | 43 |
| R15 | ACTCAAGAGCCGAGACAATCTCTGCACCATAAAACCGGGCCCGCTCCTC | 66.65 | 27 | 49 |
| F16 | GTGCAGAGATTGTCTCGGCTCTTGAGTACTTGCACTCGCGGGACGTGGTAT | 66.59 | 22 | 51 |
| R16 | GGTTTTCCAGCTTGATGTCGCGGTATACCACGTCCCGCGAGTGCAA | 66.29 | 24 | 46 |
| F17 | ACCGCGACATCAAGCTGGAAAACCTCATGCTGGACAAAGATGGCCACATCA | 66.59 | 27 | 51 |
| R17 | CTCTTTGCAGAGGCCAAAGTCAGTGATCTTGATGTGGCCATCTTTGTCCAGCATGA | 66.28 | 28 | 56 |
| F18 | GATCACTGACTTTGGCCTCTGCAAAGAGGGCATCAGTGACGGGGCCAC | 67.13 | 20 | 48 |
| R18 | CGGGGTCCCACAGAAGGTTTTCATGGTGGCCCCGTCACTGATGCC | 66.57 | 25 | 45 |
| F19 | CATGAAAACCTTCTGTGGGACCCCGGAGTACCTGGCGCCTGAGGTG | 65.9 | 21 | 46 |
| R19 | CCGGCCATAGTCATTGTCCTCCAGCACCTCAGGCGCCAGGTACTC | 65.74 | 24 | 45 |
| F20 | CTGGAGGACAATGACTATGGCCGGGCCGTGGACTGGTGGGGG | 66.85 | 18 | 42 |
| R20 | CACATCATCTCGTACATGACCACACCCAGCCCCCACCAGTCCACGGC | 66.44 | 28 | 47 |
| F21 | TGGGTGTGGTCATGTACGAGATGATGTGCGGCCGCCTGCCCTTCTAC | 66.95 | 19 | 47 |
| R21 | GAGGCGCTCGTGGTCCTGGTTGTAGAAGGGCAGGCGGCCG | 66.49 | 19 | 40 |
| F22 | CCAGGACCACGAGCGCCTCTTCGAGCTCATCCTCATGGAAGAGATCCGCTT | 66.54 | 27 | 51 |
| R22 | GCTGAGCGTGCGCGGGAAGCGGATCTCTTCCATGAGGATGAGC | 65.67 | 16 | 43 |
| F23 | CCCGCGCACGCTCAGCCCCGAGGCCAAGTCCCTGCTTG | 66.81 | 21 | 38 |
| R23 | TTGGGGTCCTTCTTAAGCAGCCCAGCAAGCAGGGACTTGGCCTCGG | 66.24 | 24 | 46 |
| F24 | TGGGCTGCTTAAGAAGGACCCCAAGCAGAGGCTTGGTGGGGGG | 66.96 | 19 | 43 |
| R24 | GACCTCCTTGGCATCGCTGGGCCCCCCACCAAGCCTCTGC | 66.6 | 21 | 40 |
| F25 | CCCAGCGATGCCAAGGAGGTCATGGAGCACAGGTTCTTCCTCAGCAT | 66.05 | 26 | 47 |
| R25 | TTCTGGACCACGTCCTGCCAGTTGATGCTGAGGAAGAACCTGTGCTCCAT | 66.2 | 23 | 50 |
| F26 | AACTGGCAGGACGTGGTCCAGAAGAAGCTCCTGCCACCCTTCAAACCT | 66.48 | 23 | 48 |
| R26 | TGTCGACCTCGGACGTGACCTGAGGTTTGAAGGGTGGCAGGAGCT | 66.55 | 22 | 45 |
| F27 | CAGGTCACGTCCGAGGTCGACACAAGGTACTTCGATGATGAATTTACCGCCC | 66.09 | 30 | 52 |
| R27 | GTCAGGGGGTGTGATTGTGATGGACTGGGCGGTAAATTCATCATCGAAGTACCTTG | 66.09 | 25 | 56 |
| F28 | GTCCATCACAATCACACCCCCTGACCGCTATGACAGCCTGGGCTTACTG | 66.06 | 24 | 49 |
| R28 | GGGTCCGCTGGTCCAGCTCCAGTAAGCCCAGGCTGTCATAGCG | 66.38 | 19 | 43 |
| F29 | GAGCTGGACCAGCGGACCCACTTCCCCCAGTTCTCCTACTCGG | 65.77 | 24 | 43 |
| R29 | GAACTGTCACTCGCGGATGCTGGCCGAGTAGGAGAACTGGGGGAAGT | 66.58 | 23 | 47 |
| F_Primer | ATGAATGAGGTGTCTGTCATCAAA | 57.79 |  | 24 |
| R_Primer | TCACTCGCGGATGCTGGCC | 65.81 |  | 19 |

**Table S4.** The oligonucleotides set designed by TmPrime for *E. coli* codon-optimized PKB2 (for gapless assembly).

| **Label** | **Oligonucleotide sequence (5’ to 3’)** | **T_m_ (°C)** | **Overlap (bp)** | **Length (nt)** |
| --- | --- | --- | --- | --- |
| F0 | ATGAATGAGGTGTCTGTCATCAAAGAAGGCTGGCTCCACAAGCGTGGTG | 64.3 | 20 | 49 |
| R0 | CGTGGCCTCCAGGTCTTGATGTATTCACCACGCTTGTGGAGCCAG | 64.9 | 25 | 45 |
| F1 | AATACATCAAGACCTGGAGGCCACGGTACTTCCTGCTGAAGAGCGACG | 63.6 | 23 | 48 |
| R1 | CCTCTCCTTGTACCCAATGAAGGAGCCGTCGCTCTTCAGCAGGAAGTAC | 65.0 | 26 | 49 |
| F2 | GCTCCTTCATTGGGTACAAGGAGAGGCCCGAGGCCCCTGATCAGAC | 64.5 | 20 | 46 |
| R2 | ACGGAGAAGTTGTTTAAGGGGGGTAGAGTCTGATCAGGGGCCTCGGG | 65.4 | 27 | 47 |
| F3 | TCTACCCCCCTTAAACAACTTCTCCGTAGCAGAATGCCAGCTGATGAAGACC | 65.6 | 25 | 52 |
| R3 | GTGTTGGGTCGCGGCCTCTCGGTCTTCATCAGCTGGCATTCTGCT | 66.1 | 20 | 45 |
| F4 | GAGAGGCCGCGACCCAACACCTTTGTCATACGCTGCCTGCAGTGG | 66.7 | 25 | 45 |
| R4 | GTGGAAGGTCCTCTCGATGACTGTGGTCCACTGCAGGCAGCGTATGACAAAG | 67.7 | 27 | 52 |
| F5 | ACCACAGTCATCGAGAGGACCTTCCACGTGGATTCTCCAGACGAGAGGGAGGAG | 67.4 | 27 | 54 |
| R5 | CCATCTGGATGGCCCGCATCCACTCCTCCCTCTCGTCTGGAGAATCCAC | 67.3 | 22 | 49 |
| F6 | TGGATGCGGGCCATCCAGATGGTCGCCAACAGCCTCAAGCAGC | 66.4 | 21 | 43 |
| R6 | GTCCTCGCCTGGGGCCCGCTGCTTGAGGCTGTTGGCGA | 65.1 | 17 | 38 |
| F7 | GGGCCCCAGGCGAGGACCCCATGGACTACAAGTGTGGCTCCC | 67.1 | 25 | 42 |
| R7 | TCCTCAGTCGTGGAGGAGTCACTGGGGGAGCCACACTTGTAGTCCATGGG | 67.3 | 25 | 50 |
| F8 | CCAGTGACTCCTCCACGACTGAGGAGATGGAAGTGGCGGTCAGCAAGG | 66.3 | 23 | 48 |
| R8 | GTCATTCATGGTCACTTTAGCCCGTGCCTTGCTGACCGCCACTTCCATC | 64.4 | 26 | 49 |
| F9 | CACGGGCTAAAGTGACCATGAATGACTTCGACTATCTCAAACTCCTTGGCAAGG | 65.0 | 28 | 54 |
| R9 | CACCAGGATGACTTTGCCAAAGGTTCCCTTGCCAAGGAGTTTGAGATAGTCGAA | 64.8 | 26 | 54 |
| F10 | GAACCTTTGGCAAAGTCATCCTGGTGCGGGAGAAGGCCACTGGCC | 65.6 | 19 | 45 |
| R10 | CGCAGGATCTTCATGGCGTAGTAGCGGCCAGTGGCCTTCTCCCG | 65.7 | 25 | 44 |
| F11 | GCTACTACGCCATGAAGATCCTGCGAAAGGAAGTCATCATTGCCAAGGATGAAGTC | 65.8 | 31 | 56 |
| R11 | GGCTCTCGGTGACTGTGTGAGCGACTTCATCCTTGGCAATGATGACTTCCTTT | 65.6 | 22 | 53 |
| F12 | GCTCACACAGTCACCGAGAGCCGGGTCCTCCAGAACACCAGGCA | 66.5 | 22 | 44 |
| R12 | CTTCAGCGCAGTGAGGAACGGGTGCCTGGTGTTCTGGAGGACCC | 66.2 | 22 | 44 |
| F13 | CCCGTTCCTCACTGCGCTGAAGTATGCCTTCCAGACCCACGACCG | 66.6 | 23 | 45 |
| R13 | GTTGGCATACTCCATCACAAAGCACAGGCGGTCGTGGGTCTGGAAGGCATA | 66.6 | 28 | 51 |
| F14 | CCTGTGCTTTGTGATGGAGTATGCCAACGGGGGTGAGCTGTTCTTCCACCT | 66.5 | 23 | 51 |
| R14 | TGTGAAGACACGCTCCCGGGACAGGTGGAAGAACAGCTCACCCCC | 67.0 | 22 | 45 |
| F15 | GTCCCGGGAGCGTGTCTTCACAGAGGAGCGGGCCCGGTTTTATG | 66.1 | 22 | 44 |
| R15 | TACTCAAGAGCCGAGACAATCTCTGCACCATAAAACCGGGCCCGCTCCTC | 66.4 | 28 | 50 |
| F16 | GTGCAGAGATTGTCTCGGCTCTTGAGTACTTGCACTCGCGGGACGTGG | 66.6 | 20 | 48 |
| R16 | GGTTTTCCAGCTTGATGTCGCGGTATACCACGTCCCGCGAGTGCAAG | 65.7 | 27 | 47 |
| F17 | TATACCGCGACATCAAGCTGGAAAACCTCATGCTGGACAAAGATGGCCACATC | 65.9 | 26 | 53 |
| R17 | TCTTTGCAGAGGCCAAAGTCAGTGATCTTGATGTGGCCATCTTTGTCCAGCATGA | 66.8 | 29 | 55 |
| F18 | AAGATCACTGACTTTGGCCTCTGCAAAGAGGGCATCAGTGACGGGGCCA | 67.5 | 20 | 49 |
| R18 | CGGGGTCCCACAGAAGGTTTTCATGGTGGCCCCGTCACTGATGCCC | 67.8 | 26 | 46 |
| F19 | CCATGAAAACCTTCTGTGGGACCCCGGAGTACCTGGCGCCTGAGGTGC | 67.6 | 22 | 48 |
| R19 | GCCCGGCCATAGTCATTGTCCTCCAGCACCTCAGGCGCCAGGTACTC | 68.7 | 25 | 47 |
| F20 | TGGAGGACAATGACTATGGCCGGGCCGTGGACTGGTGGGGGCTGG | 67.7 | 20 | 45 |
| R20 | CCGCACATCATCTCGTACATGACCACACCCAGCCCCCACCAGTCCACG | 67.6 | 28 | 48 |
| F21 | GTGTGGTCATGTACGAGATGATGTGCGGCCGCCTGCCCTTCTACAACCAGGA | 68.6 | 24 | 52 |
| R21 | GAGCTCGAAGAGGCGCTCGTGGTCCTGGTTGTAGAAGGGCAGGCGG | 67.8 | 22 | 46 |
| F22 | CCACGAGCGCCTCTTCGAGCTCATCCTCATGGAAGAGATCCGCTTCCCG | 67.9 | 27 | 49 |
| R22 | CCTCGGGGCTGAGCGTGCGCGGGAAGCGGATCTCTTCCATGAGGAT | 68.6 | 19 | 46 |
| F23 | CGCACGCTCAGCCCCGAGGCCAAGTCCCTGCTTGCTGGGCT | 67.7 | 22 | 41 |
| R23 | GCCTCTGCTTGGGGTCCTTCTTAAGCAGCCCAGCAAGCAGGGACTTGG | 67.6 | 26 | 48 |
| F24 | GCTTAAGAAGGACCCCAAGCAGAGGCTTGGTGGGGGGCCCAGCG | 67.9 | 18 | 44 |
| R24 | CTGTGCTCCATGACCTCCTTGGCATCGCTGGGCCCCCCACCAA | 67.1 | 25 | 43 |
| F25 | ATGCCAAGGAGGTCATGGAGCACAGGTTCTTCCTCAGCATCAACTGGCAGGA | 67.0 | 27 | 52 |
| R25 | GGCAGGAGCTTCTTCTGGACCACGTCCTGCCAGTTGATGCTGAGGAAGAAC | 67.5 | 24 | 51 |
| F26 | CGTGGTCCAGAAGAAGCTCCTGCCACCCTTCAAACCTCAGGTCACGTCC | 66.7 | 25 | 49 |
| R26 | TCATCATCGAAGTACCTTGTGTCGACCTCGGACGTGACCTGAGGTTTGAAGGGT | 66.2 | 29 | 54 |
| F27 | GAGGTCGACACAAGGTACTTCGATGATGAATTTACCGCCCAGTCCATCACAATCACA | 66.9 | 28 | 57 |
| R27 | AGGCTGTCATAGCGGTCAGGGGGTGTGATTGTGATGGACTGGGCGGTAAAT | 68.3 | 23 | 51 |
| F28 | CCCCCTGACCGCTATGACAGCCTGGGCTTACTGGAGCTGGACCAGC | 67.4 | 23 | 46 |
| R28 | AGTAGGAGAACTGGGGGAAGTGGGTCCGCTGGTCCAGCTCCAGTAAGCCC | 68.8 | 27 | 50 |
| F29 | GGACCCACTTCCCCCAGTTCTCCTACTCGGCCAGCATCCGCGAGTGA | 68.0 | 20 | 47 |
| R29 | TCACTCGCGGATGCTGGCCG |  |  | 20 |
| F_Primer | ATGAATGAGGTGTCTGTCATCAAAGAAGG | 63.6 |  | 29 |
| R_Primer | TCACTCGCGGATGCTGGCCG | 68.0 |  | 20 |

**Equations for DNA melting temperature calculation**

Richard Owczarzy et al [1] investigated the effects of magnesium, potassium, sodium, Tris ions, and deoxynucleoside triphosphates on melting profiles of duplex DNA oligomers and collected large melting data sets. An empirical correction function was developed that predicts melting temperatures, transition enthalpies, entropies, and free energies in buffers containing magnesium and monovalent cations. The new correction function significantly improves the accuracy of predictions and accounts for ion concentration, G-C base pair content, and length of the oligonucleotides. Their methods are adapted to calculate DNA Tm in this research. The procedure is described in detail below.

Firstly, the calculation of monovalent cations concentration [${Mon}^{+}]$：

$\left[ {Mon}^{+} \right]=\left[ K^{+} \right]+\left[ {Tris}^{+} \right]+[{Na}^{+}]$ (1)

When [${Mon}^{+}]$ is equal to zero, Equation (2) is used to compute the Tm：

$\frac{1}{Tm{(Mg}^{2+})}=\frac{1}{Tm\left( 1M{Na}^{+} \right)}+a+bln\left[ {Mg}^{2+} \right]+f_{GC}\left( c+dln\left[ {Mg}^{2+} \right] \right)+ \frac{e+fln\left[ {Mg}^{2+} \right]+g{(\ln\left[ {Mg}^{2+} \right])}^{2}}{2(N_{bp}-1)}$ (2)

The parameters for Equation (2)：

**Table S5**

| **parameter** | value($K^{-1}$) | standard error($K^{-1}$) |
| --- | --- | --- |
| a | $3.92\times{10}^{-5}$ | $0.2\times{10}^{-5}$ |
| b | $-9.11\times{10}^{-6}$ | $0.5\times{10}^{-6}$ |
| c | $6.26\times{10}^{-5}$ | $0.4\times{10}^{-5}$ |
| d | $1.42\times{10}^{-5}$ | $0.08\times{10}^{-5}$ |
| e | $-4.82\times{10}^{-4}$ | $0.7\times{10}^{-4}$ |
| f | $5.25\times{10}^{-4}$ | $0.2\times{10}^{-4}$ |
| g | $8.31\times{10}^{-5}$ | $0.2\times{10}^{-5}$ |

When [${Mon}^{+}]$ is not equal to zero, calculate the coefficient R：

$R=\frac{\sqrt{[{Mg}^{2+}]}}{[{Mon}^{+}]}$ (3)

If R < 0.22，use Equation (4)：

$$\frac{1}{Tm\left( {Mon}^{+} \right)}=\frac{1}{Tm\left( 1M{Na}^{+} \right)}+\left( 4.29f_{GC}-3.95 \right)\times{10}^{-5}\ln\left[ {Mon}^{+} \right]$$

$+9.40\times{10}^{-6}{(ln[{Mon}^{+}])}^{2}$ (4)

If R < 6.0，use Equation (2) and **Table S5**. But the parameters of *a*, *d* and *g* are calculated by below equation：

$a=3.92\times{10}^{-5}(0.843-0.352\sqrt{\left[ {Mon}^{+} \right]}\times ln[{Mon}^{+}])$ (5)

$d=1.42\times{10}^{-5}[1.279-4.03\times{10}^{-3}\ln\left[ {Mon}^{+} \right]-8.03\times{10}^{-3}{(ln[{Mon}^{+}])}^{2}$(6)

$g=8.31\times{10}^{-5}\left[ 0.486-0.258\ln\left[ {Mon}^{+} \right]+5.25\times{10}^{-3}\left( \ln\left[ {Mon}^{+} \right] \right)^{3} \right]$ (7)

If R > 6.0，use Equation（2） and the parameters of **Table S5**.

Tm of DNA duplexes in 1M [Na^+^] are calculated by Nearest-Neighbor model, which is developed by Richard Owczarzy [2]：

$$Tm=\frac{\Delta H^{\circ}}{(\Delta S^{\circ}+RlnC_{t})}$$

$\Delta H^{\circ}$ is standard enthalpy change，$\Delta S^{\circ}$ is standard entropy change，R is gas constant，its value is $1.987cal/kmol$, $C_{t}$ is the concentration of DNA duplexes. When DNA molecule is asymmetrical, the concentration of DNA duplexes is $C_{t}/4$.

The thermodynamics parameters table (**Table S6**) developed by John SantaLucia [3] is used in the equations to calculate the DNA duplexes Tm in 1M [Na^+^]:

**Table S6**

| Propagation | $\boldsymbol{\Delta H}\boldsymbol{^{\circ}}$ | $\boldsymbol{\Delta S}\boldsymbol{^{\circ}}$ | $\boldsymbol{\Delta G}_{\boldsymbol{37}}^{\boldsymbol{^{\circ}}}$ |
| --- | --- | --- | --- |
| sequence | $\boldsymbol{(kcal}\boldsymbol{mol}^{\boldsymbol{-}\boldsymbol{1}}\boldsymbol{)}$ | **(e.u.)** | $\boldsymbol{(}\boldsymbol{kcal}\boldsymbol{mol}^{\boldsymbol{-}\boldsymbol{1}}\boldsymbol{)}$ |
| AA/TT | $-7.6$ | $-21.3$ | $-1.00$ |
| AT/TA | $-7.2$ | $-20.4$ | $-0.88$ |
| TA/AT | $-7.2$ | $-21.3$ | $-0.58$ |
| CA/GT | $-8.5$ | $-22.7$ | $-1.45$ |
| GT/CA | $-8.4$ | $-22.4$ | $-1.44$ |
| CT/GA | $-7.8$ | $-21.0$ | $-1.28$ |
| GA/CT | $-8.2$ | $-22.2$ | $-1.30$ |
| CG/GC | $-10.6$ | $-27.2$ | $-2.17$ |
| GC/CG | $-9.8$ | $-24.4$ | $-2.24$ |
| GG/CC | $-8.0$ | $-19.9$ | $-1.84$ |
| Initiation | $+0.2$ | $-5.7$ | $+1.96$ |
| Terminal AT penalty | $+2.2$ | $+6.9$ | $+0.05$ |
| Symmetry correction | $0.0$ | $-1.4$ | $+0.43$ |

eg: $AC/TG$ stand for 5’ – AC – 3’ Watson-Crick complementary with 3’ – TG–5’.

**REFERENCES**

1. Owczarzy,R., Moreira,B.G., You,Y., Behlke,M.A. and Walder,J.A. Predicting stability of DNA duplexes in solutions containing magnesium and monovalent cations. *Biochemistry* **2008**, 47, 5336–5353.
2. Owczarzy, R., Vallone, P. M., Gallo, F. J., Paner, T. M., Lane, M. J., and Benight, A. S. Predicting sequence-dependent melting stability of short duplex DNA oligomers. *Biopolymers* **1997**, 44, 217–239.
3. SantaLucia,J. Jr. and Hicks,D. The thermodynamics of DNA structural motifs. Annu. Rev. *Biophys. Biomol. Struct*. **2004**, 33, 415–440.
